# The Role of Magnesium in Neurological Disorders

**DOI:** 10.3390/nu10060730

**Published:** 2018-06-06

**Authors:** Anna E. Kirkland, Gabrielle L. Sarlo, Kathleen F. Holton

**Affiliations:** 1 Department of Psychology, Behavior, Cognition and Neuroscience Program, American University, Washington, DC 20016, USA; ak0698a@american.edu (A.E.K.); gs0703a@american.edu (G.L.S.); 2 Department of Health Studies, American University, Washington, DC 20016, USA; 3 Center for Behavioral Neuroscience, American University, Washington, DC 20016, USA

**Keywords:** magnesium, excitotoxicity, glutamate, migraine, chronic pain, epilepsy, Alzheimer’s, Parkinson’s, stroke

## Abstract

Magnesium is well known for its diverse actions within the human body. From a neurological standpoint, magnesium plays an essential role in nerve transmission and neuromuscular conduction. It also functions in a protective role against excessive excitation that can lead to neuronal cell death (excitotoxicity), and has been implicated in multiple neurological disorders. Due to these important functions within the nervous system, magnesium is a mineral of intense interest for the potential prevention and treatment of neurological disorders. Current literature is reviewed for migraine, chronic pain, epilepsy, Alzheimer’s, Parkinson’s, and stroke, as well as the commonly comorbid conditions of anxiety and depression. Previous reviews and meta-analyses are used to set the scene for magnesium research across neurological conditions, while current research is reviewed in greater detail to update the literature and demonstrate the progress (or lack thereof) in the field. There is strong data to suggest a role for magnesium in migraine and depression, and emerging data to suggest a protective effect of magnesium for chronic pain, anxiety, and stroke. More research is needed on magnesium as an adjunct treatment in epilepsy, and to further clarify its role in Alzheimer’s and Parkinson’s. Overall, the mechanistic attributes of magnesium in neurological diseases connote the macromineral as a potential target for neurological disease prevention and treatment.

## 1. Introduction

Magnesium is a very important macromineral in the diet with a multitude of roles in the human body, including serving as a cofactor in more than 300 enzymatic reactions. Magnesium is essential for regulation of muscle contraction (including that of the heart), blood pressure, insulin metabolism, and is required for the synthesis of DNA, RNA, and proteins [1]. In the nervous system, magnesium is important for optimal nerve transmission and neuromuscular coordination, as well as serving to protect against excitotoxicity (excessive excitation leading to cell death) [1,2]. 

**Figure 1:**
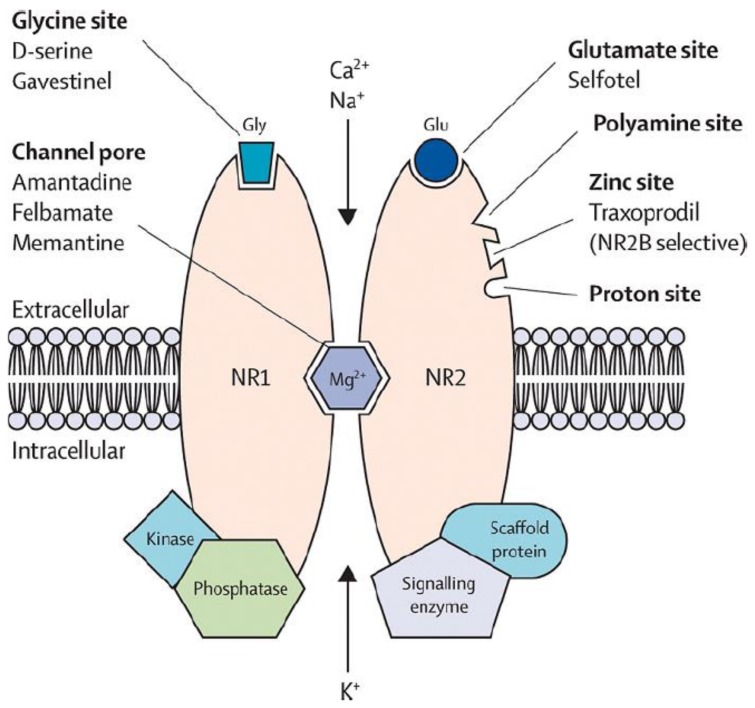
Glutamatergic *N*-methyl-d-aspartate receptor with magnesium block of calcium channel. Reprinted from [8] with permission from Elsevier.

One of the main neurological functions of magnesium is due to magnesium’s interaction with the *N*-methyl-d-aspartate (NMDA) receptor. Magnesium serves as a blockade to the calcium channel in the NMDA receptor (Figure 1), and must be removed for glutamatergic excitatory signaling to occur [3]. Low magnesium levels may theoretically potentiate glutamatergic neurotransmission, leading to a supportive environment for excitotoxicity, which can lead to oxidative stress and neuronal cell death [4]. Abnormal glutamatergic neurotransmission has been implicated in many neurological and psychiatric disorders [5] including: migraine, chronic pain, epilepsy, Alzheimer’s, Parkinson’s, and stroke, in addition to depression and anxiety, which are commonly comorbid with these neurological disorders. Molecular studies [6] and animal studies [7] have shown neuronal protection from pre-treatment with magnesium, making this mineral of intense interest for its potential neuroprotective role in humans. Thus, magnesium could be an important dietary factor in the prevention and/or treatment of the above conditions. 

It has been estimated that approximately half of the US population is consuming inadequate amounts of magnesium [9]. Due to the wide-ranging functions of magnesium, inadequate intake could predispose individuals to multiple health issues, including those related to neurological conditions. This review aims to summarize the recent human literature on what is known about magnesium and the following neurological disorders: migraine, chronic pain, epilepsy, Alzheimer’s, Parkinson’s, and stroke, as well as anxiety and depression. Recommendations will also be made for future research directions.

## 2. Materials and Methods 

**Figure 2:**
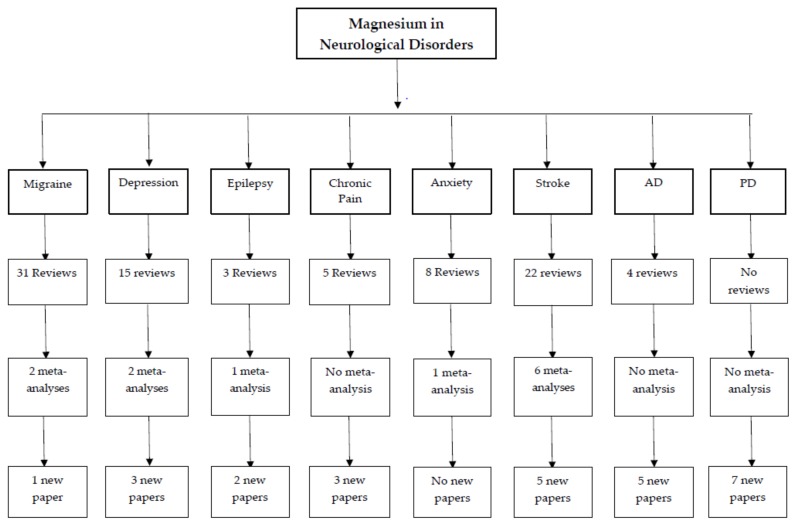
Flow chart of magnesium in neurological disorders literature. New papers refer to published research not included in previously-published reviews and meta-analyses. AD = Alzheimer’s Disease; PD = Parkinson’s Disease.

A search was completed using PubMed, MEDLINE, PsychINFO, and Wiley-Blackwell Cochrane Library. Abstracts were pulled for all available literature and were then reviewed by all three authors and a consensus decision was made about which papers met inclusion criteria. Studies of adult human populations from any year were included if they were written in English. Papers were reviewed for every article that met the review criteria, including magnesium levels (e.g., blood serum, cerebrospinal fluid, etc.) or magnesium treatment in human populations with migraine, chronic pain, anxiety, depression, epilepsy, Alzheimer’s disease, Parkinson’s disease, and stroke. Search strings included the neurological or commonly comorbid disorder AND “magnesium”, “intravenous magnesium”, “oral magnesium”, or “magnesium treatment”. Reviews and meta-analyses were used to gain an understanding of where the current literature stands in magnesium research across neurological disorders. The most recent reviews and meta-analyses are discussed in order to reduce duplication and provide a wide scope of the literature previously reviewed. New studies are described in detail to provide an update on the literature. If there was not a review or meta-analysis, like in the case of Parkinson’s disease, available research was compiled and reviewed. Figure 2 summarizes the search strategy and demonstrates the disparity in the number of magnesium research studies ranging from the most studied (i.e., migraine) to the least studied (i.e., Parkinson’s disease).

## 3. Results

### 3.1. Migraine

Migraine is the most common neurological disorder in the United Sates with a prevalence rate of 16.2% [10]. It is classified by recurrent moderate to severe headaches with or without aura, often lasting between 4 and 27 h with many associated symptoms, including nausea, vomiting, and sensitivity to various environmental stimuli [11,12]. Although the exact mechanisms are not yet fully understood, alterations in the excitability of the central nervous system, spontaneous neuronal depolarization, and abnormal mitochondria functioning have been connected to migraines. Since glutamate is the most abundant excitatory neurotransmitter, it is often linked to etiological, prevention, and treatment discussions concerning migraines [13]. Magnesium has been a proposed treatment option for migraines due to its blockade of the glutamatergic *N*-methyl-d-aspartate (NMDA) receptor, a receptor known to be an active contributor to pain transmission and cortical spreading depression [14]. Magnesium is also known to be a key metabolic factor in mitochondrial functioning and lowers membrane permeability reducing the possibility of spontaneous neuronal depression due to hyperexcitability [15]. Past research has shown that significantly lower levels of magnesium have been reported in serum, saliva, and cerebrospinal fluid of individuals with migraines during, and between, migraine attacks [12,16,17,18], along with evidence suggesting lower brain concentrations of magnesium based on MR spectroscopy [18]. 

Oral and intravenous magnesium administration has been proposed as a treatment option for migraines since the late 1980s and the results have been analyzed by several meta-analyses and reviews over the past three decades. In 2014, Choi and Parmer completed a meta-analysis using odds ratio (OR) on 5 randomized controlled trials published between 2000 and 2005 [19,20,21,22,23] and did not find strong evidence for magnesium as an effective treatment. It was concluded that i.v. magnesium resulted in a 7% lower relief rate 30 min post administration than the control groups (OR = −0.07), 37% greater side effect response rate (OR = 0.37), and no significant difference between the use of i.v. magnesium, placebo, or other migraine medications tested. However, 1 study included in the meta-analysis showed differences between migraines with and without aura; i.v. magnesium was significantly more effective at migraine relief in individuals with aura than placebo at 60 min post administration (*p* < 0.05) [24]. A more recent meta-analysis by Chiu et al. in 2016, reviewed a larger sample of randomized clinical trials with 11 studies investigating the effects of intravenous magnesium on acute migraines [19,20,21,25,26,27,28,29,30] and 10 studies investigating the effects of oral magnesium on migraine prophylaxis [16,31,32,33,34,35,36,37,38,39]. Using odds ratios, it was concluded that i.v. magnesium treatment for acute migraines resulted in significant relief across 15–45 min (OR = 0.23), 120 min (OR = 0.20), and 24 h (OR = 0.27) post magnesium administration. Similarly, oral magnesium treatment resulted in significantly reduced frequency of migraine attacks (OR = 0.20) and intensity of the attacks (OR = 0.27). Overall, this meta-analysis presents evidence for the usefulness of magnesium in both i.v. or oral forms on the treatment of migraines [40]. It also expands on the previous meta-analysis since it included randomized clinical trials published in English and Chinese investigating i.v. magnesium or oral magnesium treatment. This broadened the scope, sample size, and external validity of their work. 

One quasi-experimental study has been published since the most current review comparing 2 g of i.v. magnesium sulfate compared to 60 mg of caffeine citrate on individuals presenting with a migraine at two hospitals. While both groups displayed improved pain scores over 1 hour, the magnesium group had significantly greater improvement when compared to the group receiving caffeine (*p* < 0.001) [41]. 

The beneficial use of magnesium in the prevention of migraine and the quality of life improvement has a Grade C evidence classification, meaning it is possibly an effective treatment based on current data. This classification is based on finding a reduction in migraine days between 22–43% across five clinical trials reviewed from 1990–2016. It is suggested that 600 mg of magnesium daily may be a safe and cost effective component of migraine care [42]. While Grade C evidence is not ideal, evidence of the effectiveness for the prevention and treatment of migraines using oral or i.v. magnesium may very well become stronger over time with the publication of more intervention and prospective cohort studies. Overall, while recent evidence does point towards i.v. and oral magnesium as potentially effective treatment options, randomized controlled clinical trials with larger sample sizes and standardized experimental designs need to be conducted in order to have more confidence in the efficacy of magnesium treatment for migraines, and to better understand how magnesium compares to current pharmaceuticals used in the prevention and treatment of migraines.

### 3.2. Chronic Pain

Pain is a universal sensation that can be presented in several different forms, ranging from acute to chronic. Chronic pain is broadly defined as persistent pain lasting at least three months often spurred on by central pain amplification, although the exact mechanism of pain can vary (e.g., nociceptive, neuropathic, central, etc.) or is sometimes unidentified [43]. It is estimated that chronic regional pain may be present in 20–25% of the population and chronic widespread pain may be present in 10% of the population [44]. As discussed earlier, magnesium blocks the NMDA receptor channels limiting the influx of calcium. Therefore, moderate doses of magnesium may be able to reduce the risk of excitotoxicity [45]. It is proposed that the pain relieving effects of magnesium may be dependent on the blockade of NMDA receptors in the spinal cord [46]. Magnesium is thought to produce antinociceptive and analgesic effects in patients with chronic pain and has been studied as a treatment target for chronic pain in several forms [45].

Research exploring the analgesic use of magnesium in chronic pain disorders is limited by the type and severity of chronic pain evaluated. A review on the use of magnesium as an alternative treatment for chronic pain was recently published [47]. Chronic pain was defined as pain lasting more than three months in any body part, including chronic complex regional pain syndrome, chronic low back pain, fibromyalgia, neuropathy, or pain of vascular origin. Two double-blind randomized clinical trials on complex regional pain syndrome (CRPS) [48,49] and 1 double-blind randomized clinical trial on chronic low back pain [50] were reviewed. The studies used intravenous, intradermal, and oral magnesium administration compared to placebo. The 2 CRPS randomized clinical trials had conflicting results; Fischer et al. reported no differences in CRPS pain between patients who received i.v. magnesium and those who received a placebo using several outcome measures [48]. However, van de Plas reported significant differences between intramuscular magnesium administration and a placebo on the numeric rating scale (NRS) pain assessment scores, but not on the McGill Pain Questionnaire [49]. Both studies reported more adverse effects in the groups receiving magnesium than placebo. Yousef and Al-deeb investigated the use of i.v. magnesium followed by oral magnesium compared to a placebo on chronic lower back pain over six months using NRS pain assessment scores. Beginning at two weeks, and continuing throughout the six months of follow up, the group receiving magnesium treatment had significantly improved scores from baseline measurements. Furthermore, the magnesium group had significantly lower pain scores than the placebo group at six months [50]. Based on the review of these studies, magnesium may be a viable treatment option for some types of chronic pain. A protocol was published in 2015 describing a clinical trial that is currently investigating the effects of oral magnesium administration in patients with peripheral arterial occlusive disease [51]. This trial will add much needed evidence on the potential validity of using oral magnesium as a treatment for chronic pain.

Fibromyalgia was initially considered a rheumatic disorder, but is now known to be a neurological condition, with intense widespread pain and tenderness coupled with other unpleasant symptoms, such as severe fatigue, cognitive dysfunction, memory loss, headache, and sleep problems [52,53]. As with other chronic widespread pain conditions, it is thought that pain neurotransmission occurs through glutamate’s action on the NMDA receptor [54]; thus, magnesium is likely to play a protective role. It has also been proposed that fibromyalgia may be a result of insufficient levels of substances necessary for ATP synthesis, such as oxygen, magnesium, ADP, and inorganic phosphate. Magnesium is a key component within this process as it is needed for both aerobic and anaerobic glycolysis. It also aids in maintaining low cytosolic calcium in order to limit the inhibition of ATP synthesis within the mitochondria, ultimately reducing the chances of cell death caused by mitochondrial calcification [55]. Due to the overlap in symptomatology and the mechanistic actions of magnesium, researchers have studied magnesium levels in individuals with fibromyalgia, resulting in conflicting findings between modes of magnesium detection. Erythrocyte [56,57,58,59] and intracellular muscle magnesium levels [60] are decreased in fibromyalgia patients while there has been evidence that plasma and serum levels remain in normal ranges [57,58,59,61]. However, other studies have shown significantly decreased magnesium serum levels in fibromyalgia patients, as compared to controls [53,62,63]. Based on these findings, a recent study examined the effects of 300 mg magnesium citrate and 10 mg of amitriptyline, alone and in combination, in 60 women with fibromyalgia and 20 age- and sex-matched controls over eight weeks. Both erythrocyte and serum magnesium levels were significantly lower in the fibromyalgia group. Furthermore, the group receiving the combination of magnesium and amitriptyline reported significantly decreased pain across various pain and tenderness index scores, while magnesium alone resulted in an improvement in the number of tender points and the intensity of fibromyalgia pain [53]. Another study reported improved scores on the Fibromyalgia Impact Questionnaire-Revised (FIQR) after 8 weeks of transdermal magnesium chloride solution use [64].

It is too early to conclude whether or not magnesium is a viable treatment option for general or specific forms of chronic pain. However, the preliminary data concerning varying levels of systemic magnesium and supplementation of magnesium orally, transdermally, and intravenously for fibromyalgia and other forms of chronic pain, suggest the potential for magnesium to be an important player in the treatment and prevention of chronic pain. 

### 3.3. Anxiety and Depression

Anxiety and depression are both common comorbid conditions with neurological illness [65] especially with chronic pain conditions [66], including migraine [67]. Anxiety and depression are also similarly mediated by altered glutamatergic neurotransmission, which may account for this comorbidity [68,69]. Since magnesium has the ability to modulate glutamatergic neurotransmission through its action at the *N*-methyl-d-asparate (NMDA) receptor [70], it may be possible for hypomagnesaemia to contribute to both the neurological symptoms, as well as the psychiatric symptoms.

With a lifetime prevalence rate of 15% in the general population, anxiety is considered the most pervasive psychiatric affective disorder [71]. Magnesium is involved in several physiological processes in the psychoneuroendrocrine system and modulates the hypothalamic pituitary adrenal (HPA) axis, along with blocking the calcium influx of NMDA glutamatergic receptors, all of which help prevent feelings of stress and anxiety [72]. While the data on serum and cerebrospinal fluid levels of magnesium are limited, these concentrations have been shown to be modified by exposing individuals to various types of stress, resulting in a reduction in serum magnesium due to excretion by the kidneys [73], and increasing serum levels when magnesium is administered resulting in anxiolytic-like effects [74]. Dietary intake of magnesium was also found to have a slight inverse relationship with subjective anxiety scores in a large community-based sample [75]. However, one study found no difference between the serum magnesium concentrations in patients with Generalized Anxiety Disorders when compared to controls [76]. 

In 2017, Boyle and Dye published a review on the available studies investigating the effects of magnesium, alone or in combination, on the experience of subjective anxiety or stress (i.e., mild anxiety, premenstrual syndrome, postpartum status, and hypertension) in adult populations [72]. Eight studies were reviewed which focused on the treatment of mild anxiety with magnesium alone [77], magnesium in combination with vitamin B_6_ [78,79,80,81], magnesium with fermented cow’s drink with protein hydrolysate [82], or magnesium in combination with Hawthorn extract and California poppy [83]. Modest evidence of the beneficial use of various forms of magnesium for treatment of mild to moderate anxiety was found. However, limitations were present including the occurrence of significant placebo effects and the inability to know the exact effects of magnesium when studying multiple combined compounds. Of the 7 studies which were reviewed for anxiety associated with PMS, 5 investigated the effects of oral or i.v. magnesium administration alone [84,85,86,87] and 2 investigated the effects of magnesium in combination with vitamin B_6_ [88,89]. Despite methodological and sample selection issues presented by Boyle and Dye, the authors concluded that there is a potential positive effect of magnesium alone, and even more so in combination with vitamin B_6_ on PMS. One study was reviewed for the effects of 64.4 mg of oral magnesium on anxiety related to postpartum depression with no significant effects reported [90]. Overall, the summarized findings allow for marginal support of magnesium as a treatment for mild anxiety and anxiety with PMS, with several of the studies reviewed reporting positive outcomes when administering magnesium as the sole or adjunct treatment. 

An earlier review by Lakhan and Vieira in 2010 had a similar conclusion: magnesium administration may have a positive impact on the treatment of multiple anxiety disorders. The authors also note that many studies and clinical trials conducted do not look at the effects of magnesium alone, a comment that holds true today [91]. To our knowledge, no new magnesium studies examining the effects of magnesium on anxiety disorders have been published since the 2017 review. There is currently a strong need for methodologically sound clinical trials exploring this treatment option, as it could improve the lives of those with anxiety disorders while eliminating the negative side effects from current medications to treat anxiety. 

Depression is a psychiatric disorder that affects hundreds of millions of people around the world, with major depressive disorder (MDD) accounting for 40% of the neuropsychiatric disorders in the United States [92]. Depression is linked to poor quality of life with severe impairments and, as mentioned earlier, often presents with other comorbid disorders. While there are some beneficial biomedical and clinical therapies for depression, dietary magnesium intake could be an important adjunct treatment [93,94]. 

Restoring the balance of magnesium within patients with depression has been proposed to have anti-depressive effects by protecting brain structures associated with depression by reducing the cascade of cell death caused by excitotoxicity [95,96,97]. Magnesium may also impact depressive symptoms by interacting with the HPA system, as discussed with anxiety disorders [97,98]. As seen in several other neurological disorders, lower magnesium levels have been associated with depression. One recent study reported a negative correlation between dietary intake of magnesium and depression [93]. Studies have also observed lower cerebrospinal fluid (CSF) and serum magnesium levels in individuals diagnosed with depression as compared to controls [94] along with moderately lower levels of erythrocyte magnesium levels in patients with major depression [99]. Moreover, plasma levels of magnesium were observed to be significantly lower among those with depression, as compared to healthy controls, while also being correlated with treatment response success [100] and the severity of depressive symptoms [101,102]. However, another study reported no differences in CSF, blood, and serum magnesium levels between groups [103]. This latter finding could have been due to inaccurate assessment technique, since 99% of magnesium within the human body is located intracellularly [103]. 

There is neurobiological evidence to support magnesium supplementation as a treatment for depression, however, the results from the limited number of randomized controlled trials (RCTs) is not clear cut. In 2016, Rechenberg reviewed RCTs examining the use of magnesium in depressed populations. However, only three studies, with limited insight into the potential use of magnesium as a treatment, are discussed due to the lack of literature on the subject [94]. Bhudi and colleagues compared the use of magnesium to a placebo over three months as a neuroprotective agent for patients undergoing cardiac surgery. Depressive symptoms were reported at baseline and three months postsurgery as one of the outcomes measured. While depressive symptoms decreased, no significant differences were noted between the magnesium and placebo groups [104]. An RCT was conducted in 2007 to study the utility of oral magnesium as a treatment for newly-depressed elderly individuals with type 2 diabetes. After establishing the presence of hypomagnesemia, individuals were randomized to either the magnesium or imipramine treatment group for twelve weeks of treatment administration. At follow-up, there was no significant difference between the magnesium treatment group or the imipramine treatment group, with similar improvements in both arms of the clinical trial. Thus, magnesium performed as well as imipramine, an anti-depressant drug [101]. Lastly, Walker et al. showed no significant differences in reported depressive symptoms when administering 200 mg of magnesium or placebo daily, for two menstrual cycles, in a double-blind placebo-controlled crossover study, among individuals who typically report monthly depressive symptoms with PMS [85]. 

A meta-analysis of 11 studies was recently published examining the relationship between dietary magnesium intake and the risk of depression. Using pooled measurements of relative risk (RR) and a dose-response analysis, the authors concluded that over half of the reviewed studies supported significant effects of dietary magnesium intake in relation to a decreased risk of depression (pooled RR = 0.81) with a distinct nonlinear relationship (*p* = 0.0038) between the two factors. Furthermore, the largest risk reduction was observed with 320 mg/day of magnesium [93]. This meta-analysis adds more evidence to the theory that magnesium supplementation, either through dietary intake or other routes of administration, should be continued to be researched as a potential target for the treatment of depression. 

Three newly-published studies were found since the review by Rechenberg [94] and the meta-analysis by Li et al. [93]. One clinical trial studied the use of intravenous magnesium combined with dextrose in adults with treatment-resistant depression as compared to dextrose alone in a crossover study. The authors observed significant differences in serum magnesium levels measured at two time points: baseline compared to day 8 (the last day of administration) and day 2 compared to day 8. However, depression rating scale results were not as substantial as the only difference seen was a reduction on the Patient Health Questionnaire-9 from baseline to day 7 [105]. This study was limited by its short duration of treatment. Tarleton and colleagues performed an open-label randomized trial with 126 adults comparing 248 mg of magnesium to a placebo over six weeks, resulting in the significant improvement of depression scores within the magnesium group. In fact, improvement was noted within the first two weeks of treatment [106]. Lastly, a study of 60 patients with depression and lab-confirmed hypomagnesaemia were randomized to receive either 250 mg of magnesium or a placebo for eight weeks. Using the Beck Depression Inventory-II as an outcome measure, magnesium levels and Beck scores significantly improved in the magnesium group, as compared to the placebo group [107]. It is important to note that the amount of magnesium supplemented in these latter studies was less than the recommended dietary allowance (RDA) for adults of 310–420 mg/day. 

Unfortunately, the current research implementing magnesium as a treatment option for depression has not shown consistently significant results, although this does not mean it is not an effective treatment option [103,108]. There is a need for more well-designed randomized clinical trials and prospective studies of longer duration with adequately powered sample sizes to fully understand the effects of magnesium on depression. 

### 3.4. Epilepsy

Epilepsy is a disease classified by seizure occurrence that is believed to affect 50 million people worldwide [109,110,111]. The widespread and debilitating effects of this disorder have resulted in the investigation of treatments that fall outside the classic treatment with anti-epileptic drugs (AEDs), especially when research suggests that new AEDs are no better at significantly reducing seizures or improving prognosis than older AEDs [112,113,114]. The search for alternative treatment possibilities has directed some attention towards magnesium. Magnesium is an essential element involved in many bodily processes and, as mentioned earlier, has been found to be deficient in the modern Western diet [114,115,116]. Moreover, seizure activity has been strongly linked to excessive glutamatergic neurotransmission, thus, magnesium could potentially modulate the excitotoxicity connected to epilepsy [117]. Extracellular magnesium has been reported to reduce spontaneous spikes in seizure activity via the NMDA receptor, while also decreasing the hyperexcitability of the neuronal surface [114,118,119]. In fact, it is well known that hypomagnesaemia, itself, can cause seizure activity with more severe deficiency [120]. 

Two recent reviews [114,121] have examined the literature on magnesium and epilepsy. Both reviews stress the lack of large-scale, randomized controlled trials that are essential to gaining insight into the potential role of magnesium as a treatment for epilepsy, in general, as well as more specifically, refractory epilepsy. Refractory epilepsy is of particular interest due to its lack of response to AEDs, usually resulting in the seizures and other symptoms going untreated. 

In humans, magnesium deficiency has been found to result in seizures, as well as lower levels of magnesium being observed in epileptic patients when compared to healthy controls [114,121,122,123,124,125,126,127,128]. A recent meta-analysis that included 60 studies (40 on epilepsy and 25 on febrile seizures) found that magnesium levels were not significantly different in patients with epilepsy versus controls, or in patients with febrile seizures versus controls. Yet, that same study found that hair magnesium concentrations were significantly lower in both non-treated and treated epilepsy patients versus controls [129]. Another study reported lower magnesium levels in more severe cases of epilepsy and status epilepticus as compared to moderate and mild cases [124]. The relationship between disease severity and magnesium concentration is not an area that has been explored at length and could result in valuable information if more work focused on this potential association. 

In regards to magnesium as a treatment, magnesium supplementation has been found to be beneficial for hypomagnesaemia, a known risk factor for seizures in both infants and adults [130,131]. Other conditions associated with symptomatic seizures, such as pre-eclampsia and eclampsia, have demonstrated an improvement as a result of magnesium supplementation, as well [132,133,134]. Additionally, studies that have examined subjects with a *TRPM6* gene mutation [135,136,137], juvenile onset Alpers syndrome [138], and a case of refractory status epilepticus in a subject with a normal MRI, have also reported therapeutic benefit from the administration of magnesium supplementation [114,121,139]. 

Despite the supporting evidence from these research studies, only one randomized controlled trial has been conducted on magnesium treatment for epilepsy, which focused on infantile spasms. The study found that intravenous administration of adrenocorticotropic hormone (ACTH) with magnesium supplementation for a three week period resulted in a significant reduction in seizures compared to receiving ACTH on its own. At eight weeks post administration, the group that received the magnesium supplementation had a 79% seizure-free rate compared to only a 53% seizure-free rate of the ACTH only group [140]. While this study offers promising results, there have been no other randomized controlled studies completed since the last review in 2015. A recent study investigated the interictal total serum magnesium concentrations along with serum ionized magnesium concentrations in 104 drug-resistant epileptic individuals. Data was collected at baseline and 14 years later, with results demonstrating that 60.6% (OR = 29.19) of the sample had low interictal ionized magnesium and total serum magnesium ratio [141]. 

More randomized controlled trials are needed to better understand the potential of magnesium as a treatment option for epilepsy. Such trials, with more accurate measuring of magnesium levels, would provide greater and more specific insight into the potential role of magnesium as a treatment (or adjunct treatment) for various types of epilepsy. 

### 3.5. Parkinson’s Disease

Parkinson’s disease (PD) is a neurological disorder with symptoms such as loss of balance, muscle tension, slowed body movements, resting limb tremors, and cognitive impairment [142] caused by a selective loss of dopamine in the basal ganglia. Other factors that impact PD include mitochondrial dysfunction, oxidative stress, and protein dysfunction [143]. It has been suggested that excitotoxicity caused by excessive glutamatergic neurotransmission may mediate the dopaminergic cell loss seen in Parkinson’s disease, making modulators of excitotoxicity an area of growing research interest [144]. Human research that investigates the potential role of magnesium in PD is scarce [145]. To our knowledge, no in-depth review articles focusing on magnesium and PD in humans exist. The most recently-published study was a multicenter hospital-based case-control study in Japan that examined dietary intake of metals in patients who were found to be within six years of onset for PD. The results of the study found that higher magnesium concentrations were associated with a reduced risk of PD [146]. 

Research examining magnesium levels in PD patients have yielded mixed results. Older research from the 1960s reported no differences in serum magnesium levels in PD patients compared to controls [147]; however, this research was likely limited by the types of magnesium testing available at that time. A more recent study found similar results, with no significant differences noted in hair magnesium levels between those with and without PD. Furthermore, no association between magnesium levels and disease duration or severity was observed [142]. In contrast, a study examining cerebrospinal fluid (CSF), blood, serum, urine, and hair magnesium levels of 18 controls and 91 PD patients found that CSF magnesium levels were inversely associated with disease duration and severity. The same study also concluded that PD patients with less than a year of the disease had higher magnesium levels than PD patients with more than eight years of the disease [148]. Finally, older studies comparing magnesium levels in brain areas of PD patients versus controls found that PD patients had lower magnesium levels in the cortex, white matter, basal ganglia, caudate nucleus, and brain stem as compared to controls [149,150]. It is essential to note that these studies utilized different methods to measure magnesium concentrations, which could be a factor in the contradictory results. Future research examining the role of magnesium in PD should include measurements of magnesium concentrations in the cerebral spinal fluid (CSF) (as a measure of magnesium in the central nervous system), rather than solely in the periphery. This measurement technique may be of use in other neurological disorders as well.

In short, human research of magnesium concentrations in PD is severely lacking, despite growing evidence implicating magnesium in animal studies. There is a need for more studies in PD patients focusing on magnesium concentrations in order to get a better consensus on the relationship between magnesium and PD. These few studies have provided a small window of insight into the possible role of magnesium as a treatment for PD; however, many more studies are needed before any conclusions can be drawn. 

### 3.6. Alzheimer’s Disease

Alzheimer’s disease (AD) is a degenerative neurological disorder that is characterized by synaptic loss and cognitive impairments that include deterioration in learning and memory [1]. AD presents with accumulations of beta-amyloid and tau tangles, along with inflammation and atrophy [151]. Excitotoxicity, neuroinflammation, and mitochondrial dysfunction have all been implicated in Alzheimer’s disease [152], thus, hypomagnesaemia could further impair neuronal function. Factors related to lower magnesium availability, such as malnutrition and poor nutrient intake, are also present in AD patients [153,154], making magnesium deficiency more likely. Research suggests that ionized magnesium [155], cerebral spinal fluid (CSF) magnesium, hair magnesium, plasma magnesium, and red blood cell magnesium concentrations are significantly reduced in AD patients compared to healthy and medical controls [154]. Additionally, postmortem brain examinations of AD brains have found decreased magnesium levels compared to healthy controls [154,156,157]. Magnesium depletion has been found in the hippocampus in patients with AD, providing more evidence that magnesium may be a target of treatment [156]. 

A systematic review analyzed 13 cross-sectional studies that included AD patients and healthy controls and/or medical controls [154]. The results demonstrated that AD patients had significantly lower magnesium concentrations in CSF [158,159] and in hair samples [160,161], but no such differences were found in serum [161,162,163,164], plasma [158,165,166], or ionized/ blood cell magnesium levels [165] compared to controls. Compared to medical controls, AD patients had reduced plasma [167] and ionized blood cell magnesium concentrations [168], but no differences in serum [155,169], or CSF concentrations [168]. Meanwhile, other research has found no differences between hair and serum magnesium levels in patients with AD compared to controls [170]. Hence, current research has found contradictory results on magnesium concentration levels and, thus, requires further investigation and standardized ways to measure magnesium levels. One specific study worth noting reported that ionized magnesium levels were significantly associated with cognitive function, but not with physical function, when comparing AD patients to age-matched controls [155]. Similar to Parkinson’s disease, future AD research should include a greater focus on CNS magnesium concentrations from CSF measurements, as well as using magnetic resonance spectroscopy. The above findings suggest that AD patients may be commonly deficient in magnesium.

While previous reviews have demonstrated a link between magnesium deficiency and AD in humans, there is a distinct lack of research that looks at magnesium as a treatment for AD in humans. Research to date has focused mainly on dementia, as opposed to AD specifically, and has had mixed findings. One study observed low magnesium levels in patients with mild cognitive impairment and AD patients as compared to controls [171]. Yet, another study found that both low and high magnesium concentration levels were associated with a greater risk of all-cause dementia [172]. This is the only study that provides evidence for both high and low magnesium levels being related to the risk of dementia development. As the study insists, the replication of findings from large population-based research is essential for a better understanding of these findings. For dementia, focusing on magnesium treatment through diet has been shown to improve memory [173]. The PATH through Life Project found that higher magnesium intake was related to a reduced risk of developing mild cognitive impairment and mild cognitive disorders [173]. Similarly, one study in Japan found that the greater the magnesium intake, the lower the rates of all-cause dementia and vascular dementia. However, the same relationship was absent for AD patients [174]. It is important to remember that dementia has multiple causes, including that stemming from vascular origin. Magnesium is unique for its ability to affect vascular function in addition to neuronal function [1]. Thus, magnesium may be affecting cognitive function in multiple distinct ways. Future clinical trial research is needed in this area to add to the literature in examining whether magnesium should be an adjunct treatment option in AD and/or in other types of dementia.

### 3.7. Stroke

Stroke is a cerebrovascular accident that presents itself with symptoms such as slurred speech, paralysis/numbness, and difficulty walking. Stroke can be broken down into two types, ischemic (where blood flow is impeded, usually by a clot) and hemorrhagic (where a blood vessel ruptures, causing impaired blood flow in the brain) [175]. The induced hypoxia causes excitotoxicity and resultant cell death [176]. Magnesium’s dual role in its ability to affect vascular function [177], as well as its ability to protect against excitotoxicity mediated by NMDA receptors [176], has made it an element of interest within the stroke research community. Studies examining risk of stroke and magnesium levels have yielded mixed results. Research has found no relationship between serum ionized magnesium levels and stroke risk, when based on ischemic stroke cases over a 16 years follow-up [178]. Similarly, another study reported that plasma magnesium levels were not associated with the risk of ischemic stroke in women, yet those with low ionized magnesium levels (<0.82 mmol/L) had a 57% higher risk of ischemic stroke, after controlling for potential confounds [179]. Yet, more recent studies have found that higher serum magnesium concentrations at the time of hospital admission were independently related to lower hematoma volume and lower intracerebral hemorrhage scores in patients with acute spontaneous intracerebral hemorrhage [180]. In a more general study of adults in the United States, very low serum magnesium concentrations were significantly related to increased risk of stroke mortality [181]. While magnesium levels and stroke risk have resulted in contradictory results, there is a clear suggestion of magnesium being protective against stroke.

Most of the research conducted on the association between risk of stroke and magnesium can be found in American, European, and Asian prospective cohort studies [182,183]. A recent publication on stroke reviewed multiple meta-analyses and reported a dose-dependent protective effect of magnesium against stroke [182]. Most of the meta-analyses reviewed found that each 100 mg/day increment of dietary magnesium intake provided between 2% and 13% protection against total stroke [183,184,185,186]. A recently updated meta-analysis by Fang et al. included 40 prospective cohort studies and found 22% protection against the risk of stroke when comparing people with the highest to the lowest categories of dietary magnesium intake [183]. These meta-analyses are noted in the review paper as exhibiting high homogeneity, low publication bias, and were careful to adjust for potential confounds [182]. Hence, these studies are highly reliable and support the notion of an inverse-dose dependent relationship between dietary magnesium intake and risk for total stroke. These meta-analyses suggest that increased magnesium intake, as well as higher levels of serum magnesium, appear to be beneficial in reducing total stroke risk. 

It is important to note that there are many types of stroke, and that not all stroke subtypes have demonstrated a universal relationship with magnesium. For example, one meta-analysis [184] included 7 prospective studies and found that magnesium intake levels were inversely related to the risk of ischemic stroke [178,187,188,189]; however, this inverse relationship was absent for intracerebral hemorrhage or subarachnoid hemorrhage [184,187,188]. A later study investigating whether magnesium could reduce the risk of delayed cerebral ischemia in patients with aneurysmal subarachnoid hemorrhage, also found that magnesium was not beneficial and did not reduce the risk [190]. Such evidence suggests the possibility that different types of stroke have different relationships to magnesium, an important distinction to be made for understanding magnesium’s role in the risk of stroke development. 

Investigations on magnesium levels in stroke patients have suggested an association between low magnesium levels and poor outcomes post-stroke. A recent study confirms this notion, providing evidence that low serum magnesium levels at the time of hospital admission were independently related to in-hospital mortality of patients with acute ischemic stroke [191]. An earlier study reported that decreased CSF magnesium levels were observed in ischemic stroke patients compared to controls, and that a positive association existed between low CSF magnesium levels and mortality after 7 days [192]. In addition to ischemic stroke, an observational cohort study involving patients presenting with intracerebral hemorrhage found that three month post-observation, poor functional outcomes were associated with low magnesium levels at the time of hospital admission, even after adjusting for age and measures of disease severity. Furthermore, initial and final hematoma volumes, as well as hematoma growth, were all independently and inversely correlated with low admission serum magnesium levels [193]. It is important to address the major issue when studying hypomagnesemia in stroke patients, which is that the levels are measured post diagnosis, so a causal relationship cannot be assumed and consequently there is the possibility that the lower magnesium levels are a result of stroke and not a cause of stroke [182]. 

Despite what appears to be a protective effect of magnesium levels on stroke, magnesium as a treatment for stroke has yielded less clear results. A trial investigating acute stroke has found that intravenous magnesium administration within 12 h of stroke onset does not improve death or disability outcomes [194]. Similarly, a meta-analysis also reported no beneficial effect of magnesium on delayed cerebral ischemia when started early after an aneurysmal subarachnoid hemorrhage based on 5 trials [195,196,197,198,199,200]. 

Some studies have found magnesium sulfate to be beneficial for managing post-stroke outcomes [201,202]. A meta-analysis by Chen and Carter [203] investigated 8 controlled clinical trials, 4 of which provided evidence that magnesium sulfate reduces the risk of poor outcomes 3–6 months after aneurysmal subarachnoid hemorrhage when compared to controls [198,199,201,204]. The other studies reported that magnesium sulfate was not beneficial for treating aneurysmal subarachnoid hemorrhage [199,205,206]. The overall findings of the meta-analysis suggested that magnesium sulfate may be able to decrease the risk of poor functional outcome in subarachnoid hemorrhage.

In terms of overall stroke, Panahi et al. reviewed studies that demonstrate how magnesium sulfate has been found to improve scores on different measurement indices [196,201,207], neuroprotective properties [208,209], hospital stay length [207,210], and recovery outcomes [206,207]. An additional compound that has been studied as a potential treatment for stroke is an enriched salt that is made up of a combination of magnesium and potassium. In a double-blind randomized controlled trial study, the compound was found to improve neurologic deficits following a stroke when administered for a six month period [211]. 

In summary, there is research to suggest the use of magnesium to improve outcomes post-stroke. More research is needed to understand the potential protective role of maintaining adequate magnesium levels in the prevention of stroke occurrence. 

## 4. Conclusions

In conclusion, the amount of quality data on the association of magnesium with various neurological disorders differs greatly. There is strong data for the role of magnesium in migraine and depression. There is also good potential for magnesium to be having an effect in chronic pain conditions, as well as commonly comorbid psychiatric disorders, such as anxiety and depression. Much more research is needed in regards to magnesium’s effects on epilepsy, including clinical trials evaluating the use of magnesium as an adjunct treatment. Neurological disorders, like Parkinson’s and Alzheimer’s, would benefit greatly from additional research that include measures of CNS magnesium levels (via CSF measurements and MRS). Finally, there is some research to suggest a positive effect of magnesium for improving post-stroke outcomes, and as an important dietary strategy for potentially preventing stroke, though more prospective studies are needed in this regard.
